# Beyond Coronary Stenosis: Coronary Computed Tomography Angiography and the New Approach to the Coronary Atheroma Assessment

**DOI:** 10.3390/jcm15218513

**Published:** 2026-11-02

**Authors:** Mario De Michele, Carlo Tedeschi, Carlo Liguori, Salvatore Severino, Vincenzo Tuccillo Castaldo, Pasquale Campana, Roberto Franco Enrico Pedretti, Luigi Argenziano, Stefano Nardi, Vittoria Marino, Milena Sidiropulos, Michele Clemente, Emanuela Clemente, Pasquale Guarini, Edvige Palazzo Adriano, Laura Adelaide Dalla Vecchia

**Affiliations:** 1 Division of Cardiology, S. Giuseppe Moscati Hospital, 81021 Aversa, Italy; demic@libero.it (M.D.M.); dr.vincenzocastaldotuccillo@gmail.com (V.T.C.); 2 IRCCS SYNLAB SDN, Via Crispi 2, 80100 Naples, Italy; carlo.tedeschi@hotmail.it; 3 Department of Radiology, Monaldi Hospital, A.O.R.N.dei Colli, 80100 Naples, Italy; c.liguori@gmail.com; 4 Department of Cardiology, S. Michele Private Clinic, 81024 Maddaloni, Italy; dr.sseverino@gmail.com; 5 Department of Cardiology, Pineta Grande Hospital, 81030 Castel Volturno, Italy; campanapasquale@gmail.com (P.C.); luargenz@gmail.com (L.A.); nardi.stefano@libero.it (S.N.); marinovittoria723@gmail.com (V.M.); 6 SICOA Centro Studi, IRCCS Istituti Clinici Scientifici Maugeri, 20100 Milan, Italy; roberto.pedretti@unimib.it; 7 Department of Cardiology, S. Maria delle Grazie Hospital, 80078 Pozzuoli, Italy; milesidi@gmail.com; 8 Department of Cardiology, Ospedale Madonna delle Grazie, 80078 Matera, Italy; michele.clemente@asmbasilicata.it; 9 Department of Cardiology, Policlinico “Riuniti”, 71100 Foggia, Italy; emanuela.clemente@unifg.it; 10 Department of Cardiology, Sanatrix Clinic, 80100 Naples, Italy; guarini@iol.it; 11 Department of Cardiology, Istituti Clinici Scientifici Maugeri IRCCS, Via Camaldoli 64, 20138 Milan, Italy; edvige.palazzoadriano@icsmaugeri.it

**Keywords:** coronary computed tomography angiography, cardiovascular risk, coronary atherosclerosis, coronary stenosis, cardiovascular imaging, cardiovascular prevention, high-risk plaque, coronary angiography, non-obstructive coronary disease, multidisciplinary team

## Abstract

Coronary computed tomography angiography (CCTA) has profoundly changed the diagnostic approach to patients with suspected coronary artery disease (CAD). Originally developed as a non-invasive technique for identifying or ruling out obstructive coronary stenoses, CCTA has progressively evolved into an imaging modality capable of providing a comprehensive assessment of coronary atherosclerosis. A growing body of evidence has demonstrated that the clinical value of CCTA extends far beyond the anatomical evaluation of coronary stenosis. Modern CCTA allows for the quantification of the burden of atherosclerotic plaques, the characterization of plaque composition, the identification of high-risk plaque features, and a more accurate stratification of cardiovascular risk, moving from the concept of ischemia to that of atheroma. In addition to its role in diagnosing CAD and evaluating coronary stents and bypass grafts, CCTA is also becoming increasingly important in procedure planning, cardiovascular prevention, and the personalization of treatment strategies. Overall, this could finally represent a unique opportunity to reduce the cardiovascular burden that still weighs on health care systems. Looking ahead, the growing use of CCTA in clinical practice is creating new diagnostic, interpretive, and organizational challenges. This perspective aims to discuss the evolving role of CCTA and the necessary changes regarding the new competencies required of clinical cardiologists, interventional cardiologists, and cardiovascular radiologists, taking into account the need to redefine technological standards, multidisciplinary training programs, as well as the organization and sustainability of healthcare—including environmental impact and reimbursement models—in order to fully and fairly harness the potential of modern CCTA.

## 1. Introduction

Coronary computed tomography angiography (CCTA) represents one of the innovations that has profoundly transformed the diagnostic pathway of ischemic heart disease. Owing to its high sensitivity and excellent negative predictive value, the technique was initially established as a non-invasive alternative to invasive coronary angiography (ICA) for identifying or ruling out significant coronary stenoses in patients with suspected coronary artery disease (CAD) [1,2]. For many years, the clinical value of CCTA was primarily based on its agreement with ICA, as the anatomical gold standard for evaluating coronary lesions [3]. CCTA was largely interpreted using a stenosis-focused approach, and CCTA reports were primarily centered on the degree of luminal narrowing and the identifications of lesions requiring invasive confirmation and coronary revascularization. The original goal was mainly to reduce the need for unnecessary ICA and to improve the selection of patients for further diagnostic testing or coronary revascularization. However, CCTA and ICA provide complementary rather than interchangeable information. While ICA primarily evaluates the coronary lumen, CCTA simultaneously assesses the lumen, the vascular wall, and the atherosclerotic plaque. Discrepancies may arise between the two techniques, which should not necessarily be taken as diagnostic errors. For instance, CCTA may overestimate stenosis because of calcium blooming, motion artifacts, suboptimal image quality, or small vessel caliber, whereas coronary angiography may identify lesions not adequately visualized by CCTA, particularly in distal vessels or in the presence of complex coronary anatomy. For the effective clinical use of these two techniques, it is necessary to consider all technical and patient-related factors, as well as technological requirements, including radiation dose, contrast agent dosage, patient preparation protocols, image post-processing, and potential pitfalls and artifacts.

## 2. Methodology

This narrative, non-systematic perspective was primarily based on literature published during the last ten years, supplemented by landmark studies and current guidelines and consensus documents. Relevant evidence was identified through MEDLINE, EMBASE, Scopus, and Web of Science for English-language sources using terms related to CCTA, coronary atherosclerosis, plaque burden and composition, high-risk plaque, computed tomography-derived fractional flow reserve (CT-FFR), pericoronary adipose tissue, artificial intelligence (AI), therapeutic monitoring, and technological and organizational aspects. Priority was given to guidelines, randomized trials, prospective studies, large observational cohorts, and systematic reviews. The manuscript was developed by a multidisciplinary group with expertise in clinical cardiology, interventional cardiology and cardiovascular imaging through iterative review and discussion. Disagreements were resolved by discussion and revision; no formal Delphi process or voting was performed. The working group was brought together by the scientific society SICOA (Società Italiana Cardiologia Ospedalità Accreditata) with the aim of defining the current and evolving role of CCTA. The board considered the many aspects related to CCTA: on the one hand, the need to develop new skills, redefine technological standards, and establish multidisciplinary training programs; on the other hand, the organizational management of the healthcare system as a whole, the use of available resources, the sustainability of healthcare, and the environmental impact. The article reflects the authors’ interpretation of the evidence and does not constitute an official SICOA position statement or clinical guideline but rather a call to action to consider CCTA from a broader perspective.

## 3. CCTA Perspective

### 3.1. The Oculostenotic Reflex in the Era of CCTA

Historically, the concept of “oculostenotic reflex” has described the tendency to regard any stenosis detected by ICA as an automatic indication for revascularization [4,5]. This approach has been progressively challenged by studies demonstrating that the severity of luminal narrowing in itself is not sufficient to justify an invasive treatment strategy in patients with stable CAD [6,7]. The ISCHEMIA study challenged the simple “ischemia-to-revascularization” paradigm, demonstrating that, in selected patients with stable CAD and moderate-to-severe ischemia, an initial invasive strategy did not result in a reduction of ischemic cardiovascular events or mortality compared with an initial conservative strategy [8]. The increasing use of CCTA may generate a paradoxical, undesirable “upstream oculostenotic reflex,” whereby visualization of a coronary stenosis would trigger further functional testing, ICA, or referral for intervention without adequately integrating the overall clinical context. Indeed, in a multicenter UK cohort of 5293 patients undergoing CCTA, 8% were referred to additional functional testing and 12% to ICA; importantly, 48% of these latter patients were not eligible for revascularization [9]. Data from European registries have similarly highlighted variability in subsequent referrals, despite a comparable prevalence of CAD, with greater use of non-invasive tests, ICA, and surgery in non-academic centers compared to academic centers [10]. Although these observations do not prove the existence of an imaging-induced oculostenotic reflex, they do suggest that anatomical findings may lead to potentially avoidable tests. To overcome this new pitfall, CCTA findings should be integrated with clinical history, pre-test probability, atherosclerotic burden, and, when appropriate, functional information. The DISCHARGE study demonstrated that, in patients with stable chest pain already referred for ICA, an initial CCTA strategy yielded similar major cardiovascular outcomes, with fewer procedure-related serious complications [11], while the PRECISE study demonstrated greater diagnostic efficiency by using risk-based deferred testing in low-risk patients and CCTA with selective CT-FFR in the remaining patients [12].

### 3.2. From Ischemia to Atherosclerosis: A Paradigm Shift

Over the past few decades, our understanding of CAD has evolved substantially. Traditionally, the diagnostic pathway followed a linear sequence in which symptoms or inducible ischemia prompted the search for significant coronary stenosis, which was considered the primary indication for coronary revascularization [13]. Accordingly, the prevailing paradigm could be summarized as ischemia → stenosis → revascularization.

A growing body of clinical and imaging evidence has demonstrated that cardiovascular risk depends not only on the severity of a single stenosis but also on the overall characteristics of coronary atherosclerosis [14]. Many acute coronary syndromes originate from plaques that are not severely obstructive, whereas apparently significant stenoses may remain clinically stable for years [15]. This has gradually shifted the focus from simple luminal narrowing and ischemia toward characterizing the underlying atherosclerotic disease. The recent Lancet Commission on Rethinking Coronary Artery Disease formalized this transition as a shift “from ischemia to atheroma”, conceptualizing coronary atherosclerosis as a pathological continuum that precedes obstructive stenosis, ischemia, and clinical events [16]. This approach is also consistent with current European guidelines, which integrate the anatomical assessment of both obstructive and non-obstructive CAD with clinical probability, functional information, and preventive risk assessment, rather than relying exclusively on the severity of stenosis [17]. The emerging paradigm can therefore be summarized as follows: atherosclerosis → plaque burden and biological vulnerability → cardiovascular risk → personalized prevention and treatment [18].

### 3.3. From Stenosis to Plaque: Modern CCTA Insights

**Figure 1:**
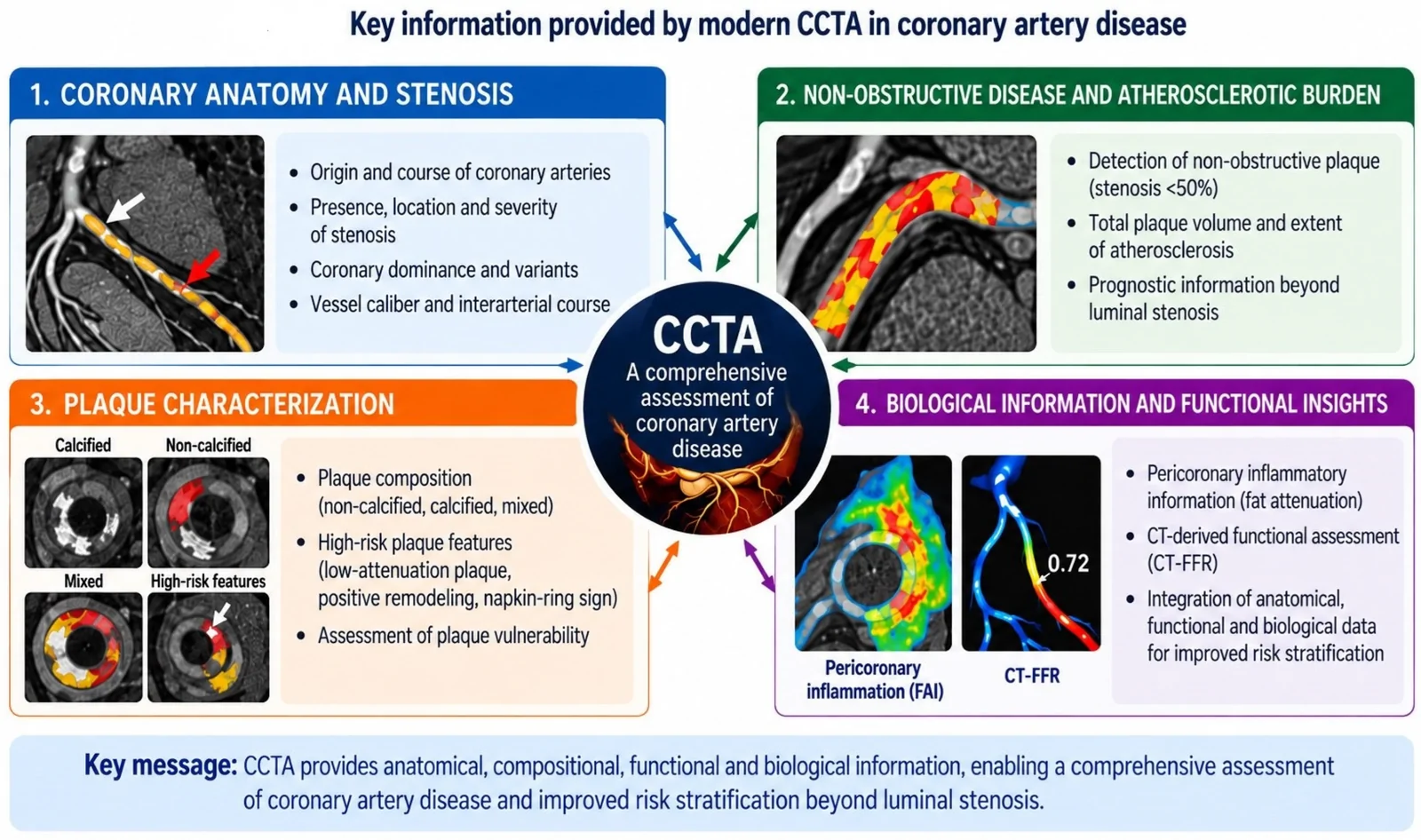
Description of the main areas of information obtainable with CCTA: coronary anatomy and stenoses, non-obstructive pathology, atherosclerotic burden, plaque characterization, and high-risk features, as well as new biological and functional insights, such as pericoronary inflammation and CT-FFR. CCTA, coronary computed tomography angiography; FAI, fat attenuation index; CT-FFR, computed tomography-derived fractional flow reserve.

One of the most significant advances in CCTA has been the progressive expansion of the clinical information obtained from a single examination capable of assessing the presence, extent, and characteristics of coronary atherosclerosis [19,20,21,22] (Figure 1).

**Figure 2:**
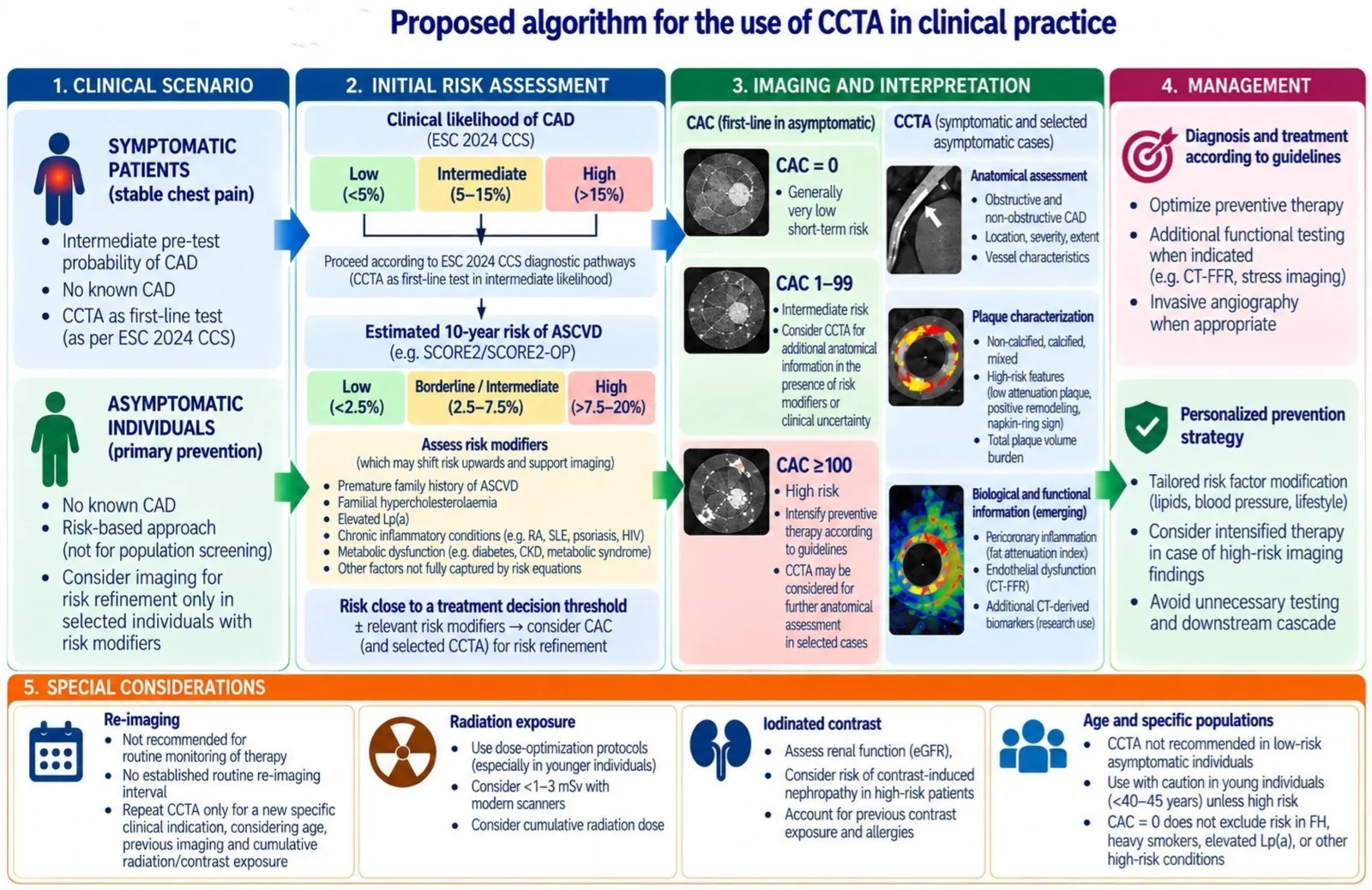
Proposal of a practical algorithm based on the patient’s clinical presentation—whether asymptomatic or symptomatic—that incorporates risk assessment and the pivotal role of CCTA, ultimately leading to the definition of a personalized treatment plan. Specific considerations are highlighted at the bottom. CAD, coronary artery disease; ASCVD, atherosclerotic cardiovascular disease; CCTA, Coronary computed tomography angiography; CT-FFR, computed tomography-derived fractional flow reserve; CAC, coronary artery calcium score; SCORE2, Systematic Coronary Risk Estimation 2; SCORE2-OP, Systematic Coronary Risk Estimation 2-Older Persons; Lp(a), lipoprotein(a); RA, rheumatoid arthritis; SLE, systemic lupus erythematosus; HIV, human immunodeficiency virus; CKD, chronic kidney disease; eGFR, estimated glomerular filtration rate, FH, familial hypercholesterolemia.

Indeed, a key contribution of CCTA is the evaluation of non-obstructive CAD [23,24,25]. Lesions causing a luminal narrowing <50% have historically been considered of limited clinical relevance, particularly in the absence of inducible ischemia. However, it has been demonstrated that even a non-obstructive plaque is associated with an increased risk of future cardiovascular events. Cardiovascular risk increases progressively with total plaque burden, the extent of coronary involvement, and plaque composition, allowing CCTA to provide continuous rather than dichotomous prognostic information across the entire spectrum of coronary atherosclerosis [26,27,28]. Another key strength of CCTA is plaque characterization, i.e., the identification of high-risk plaque features, including low-attenuation plaque, positive remodeling, spotty calcifications, and the napkin-ring sign. These imaging findings reflect biologically active plaque phenotypes and have consistently been associated with an increased risk of cardiovascular events, even in the absence of severe luminal stenosis [29,30,31,32,33,34,35]. This observation supports the notion that many acute coronary syndromes originate from vulnerable plaques rather than from the most obstructive lesions. CCTA can provide additional information on risk in selected individuals for whom conventional risk assessment would not adequately support a clinically relevant treatment decision. From an operational standpoint, this includes patients whose estimated risk is close to the therapeutic decision threshold and in whom established risk modifiers—such as a family history of premature death, elevated lipoprotein(a) levels, chronic inflammatory conditions, or other factors not fully accounted for by conventional risk equations—could lead to a clinically significant reclassification of risk [36]. CCTA should be considered only in selected cases where further characterization of coronary atherosclerosis is expected to influence therapeutic management [37,38,39,40] and should not be used as a population-based screening strategy. Traditional risk scores estimate the probability of future cardiovascular events based on clinical variables, not providing a direct visualization of coronary atherosclerosis [41,42]. The coronary artery calcium score (CAC) provides valuable information on the burden of calcified plaques, but it neither detects non-calcified plaques nor characterizes their phenotype [43,44,45,46,47,48]. CCTA provides complementary information by directly visualizing coronary atherosclerosis, quantifying plaque burden, and identifying high-risk plaque characteristics. [49]. Accordingly, traditional risk factors, CAC, and CCTA should be considered complementary components of cardiovascular risk assessment rather than competing strategies (Figure 2).

In turn, the identification of diffuse coronary atherosclerosis, non-calcified plaque, or high-risk plaque features may support intensification of preventive therapy through optimization of lipid-lowering treatment, stricter control of cardiovascular risk factors, improved treatment adherence, and individualized follow-up. Conversely, the absence of coronary atherosclerosis may support a less intensive preventive approach within the context of the patient’s overall clinical profile. In this way, CCTA complements clinical judgment, shifting cardiovascular prevention from a purely probabilistic model to one based on direct visualization of coronary atherosclerotic involvement.

An emerging application of CCTA is the assessment of treatment-related changes in coronary atherosclerosis. Serial CCTA can quantify changes in non-calcified plaque burden, calcification, and plaque composition. Studies on intensive lipid-lowering therapies have demonstrated treatment-associated changes consistent with plaque stabilization [49,50,51,52,53,54]. However, these CCTA-derived changes are imaging endpoints and not validated surrogate markers of cardiovascular outcome benefit. Completed mechanistic imaging studies, including the EVAPORATE, provided evidence of treatment-associated plaque modification [55], whereas studies evaluating newer therapies, including the VICTORION-PLAQUE and T-Plaque, should be interpreted according to their ongoing or imaging-endpoint design [53,54,55,56,57,58]. Accordingly, serial CCTA should currently be regarded primarily as a research tool for assessing the biological effects of therapy rather than as a routine method for monitoring treatment response or guiding therapeutic adjustment. Although imaging-defined plaque burden and progression may potentially contribute to more personalized prevention [59], to date no randomized trial has demonstrated that adjusting preventive therapy based on the results of subsequent CCTA improves cardiovascular outcomes compared with the care currently recommended by the guidelines.

Despite this evidence, special care must be taken to avoid overestimating the role of CCTA and overusing it. Current data support its diagnostic and prognostic value [60,61], but do not justify its use as a universal screening tool in asymptomatic individuals. There is still a lack of robust randomized prospective data demonstrating the superiority of CCTA-guided prevention, including CAC, over current risk-based strategies [62]. CCTA should be considered an advanced tool for risk reclassification and personalized prevention in carefully selected patients. The prognostic potential of CCTA in primary prevention was recently evaluated in the SCAPIS study, a population-based observational cohort comprising Swedish men and women aged 50 to 64 years. Among the 24,791 participants with no history of cardiovascular disease, measures of coronary atherosclerosis derived from CCTA provided modest incremental predictive value for first coronary events beyond traditional risk factors and CAC during a median follow-up of 7.8 years [63]. However, the SCAPIS study did not include an interventional or CCTA-guided treatment arm. Therefore, these results demonstrate incremental prognostic information rather than a clinical benefit derived from a CCTA-guided preventive strategy. Their generalizability beyond a middle-aged Swedish population also requires further evaluation.

Once again, the greatest value of CCTA was not simply the detection of obstructive stenoses, but the quantification of the overall coronary atherosclerotic burden and the identification of non-calcified plaques, both of which are associated with an increased risk of first coronary events. In summary, the strength of CCTA lies in its ability to integrate diagnostic, prognostic, and therapeutic information within a single examination. Beyond detecting coronary stenosis, it enables the assessment of non-obstructive disease, the quantification of plaque burden, the identification of vulnerable plaque, the cardiovascular risk reclassification, and the longitudinal monitoring of therapeutic response.

In this perspective, the CCTA integrates clinical risk scores, CAC, and functional imaging, transforming cardiovascular assessment from a probabilistic estimate of risk to a direct and personalized characterization of coronary atherosclerotic disease.

### 3.4. The Impact of Technology on Information Quality

The growing use of CCTA in clinical practice has highlighted an often-overlooked issue: CCTA can provide information of varying levels of detail and significance. While clinical indications and interpretation criteria are clearer, diagnostic performance may be subtly influenced by scanner technology, acquisition protocols, post-processing capabilities, and the expertise of the imaging center. Over the past decade, technological advances have significantly improved image quality and reduced many of the limitations due to several factors, such as higher detector coverage, improved spatial and temporal resolution, dual-source CT systems, and advanced iterative reconstruction algorithms [64,65,66,67]. However, these innovations are not uniformly available. In clinical practice, centers equipped with state-of-the-art technology still coexist with facilities that use older scanners or software not specifically designed for advanced cardiovascular imaging. As a result, the diagnostic accuracy of CCTA—including the evaluation of complex coronary anatomy, plaque characterization, and prognostic assessment—may vary considerably from one center to another. Equally important, updated technology makes it possible to substantially reduce radiation exposure and the volume of contrast medium used through the use of low-voltage tube protocols, ECG-triggered imaging, and advanced reconstruction algorithms. Emerging technologies, such as photon-counting CT and deep-learning-based image reconstruction, are expected to further improve image quality while reducing radiation dose [68]. Given the increasingly important role of CCTA in the diagnosis and prevention of cardiovascular diseases, it is useful to establish a quality framework for centers that perform CCTA, covering the key technological, procedural, interpretive, and safety aspects necessary to ensure high-quality practice (Table 1). Specific acquisition parameters should follow current technical recommendations and be adapted to the individual clinical context [66,67].

As for other advanced cardiovascular procedures, standards regarding scanner performance, acquisition protocols, post-processing software, and quality assurance may help ensure consistent diagnostic quality across different institutions. The challenge for the coming years will therefore consist not only in improving CCTA technology, but also in ensuring equitable and appropriate access to high-quality imaging through an adequate healthcare system, referral networks, and shared technological standards, taking into account the overall clinical, economic, and environmental implications. Only under these conditions will it be possible to consistently translate the full clinical potential of modern CCTA into daily practice.

The increasingly detailed information provided by CCTA must also be evaluated in light of potential harms and downstream consequences. Although current acquisition protocols have substantially reduced radiation exposure, the cumulative dose remains a significant factor in the case of repeated examinations. CCTA also requires the use of iodinated contrast media, making renal function and previous reactions to contrast media important factors to consider [17]. Technical limitations, including extensive coronary calcification and uncontrolled or irregular heart rates, can compromise image quality or render the examinations non-diagnostic. Furthermore, incidental extracardiac findings may lead to additional imaging studies and diagnostic procedures. Finally, the detection of non-obstructive plaques, particularly in asymptomatic individuals, should not automatically lead to the performance of functional tests, ICA, or serial CCTA. Appropriate patient selection and the avoidance of unnecessary follow-up investigations are therefore essential to prevent a new, imaging-induced upstream oculostenotic reflex.

### 3.5. The Challenge of Sustainable Cardiovascular Care: Balancing the Implementation of Value-Based Health Care with the Reduction of the Carbon Footprint of Diagnostic Testing

The implementation of Value-Based Health Care (VBHC) is an increasingly important healthcare issue and a priority for policymakers, providers, and payers alike, with the aim of maximizing health outcomes relative to the cost of care delivered [69]. For VBHC to be effective, both standardized methods for measuring outcomes and awareness of the carbon footprint generated in each process are essential. For CCTA, robust comparative data on the various commonly used imaging modalities, as well as data on the carbon emissions associated with their use, are essential. We should all be aware that the healthcare sector, globally, is responsible for approximately 4.4% of annual greenhouse gas emissions [70,71]. Available studies suggest substantial differences in the carbon footprint of cardiovascular diagnostic modalities, with echocardiography generally associated with the lowest environmental impact, CT/CCTA with an intermediate footprint, and nuclear diagnostics such as single-photon emission tomography (SPECT) with a higher footprint, while invasive/interventional procedures may generate substantially higher emissions [72,73]. However, direct comparisons must be interpreted with caution, as published estimates use heterogeneous methodologies and differ regarding the inclusion of scanner energy consumption, radiopharmaceutical production, consumables, patient and staff travel, and waste. In particular, reliable comparative life-cycle assessments that specifically compare complete diagnostic pathways based on CCTA, SPECT, stress echocardiography, and ICA remain limited. Given the need for rapid decarbonization, the contribution of researchers and clinicians is essential to provide clear guidance and ensure appropriate prescriptions for diagnostic imaging tests. Administrators also play an equally important role, as they will need to choose when to purchase new scanners and opt for models with low standby power consumption, as well as turning scanners off, where possible, outside of working hours. The “One Health” logic is proving to be increasingly necessary in health policies in order to achieve a balanced and effective health system; therefore, it must also be taken into account in the management of diagnostic-therapeutic pathways in the field of cardiovascular prevention [74].

### 3.6. The Challenge of Education: New Information Requires New Skills

**Figure 3:**
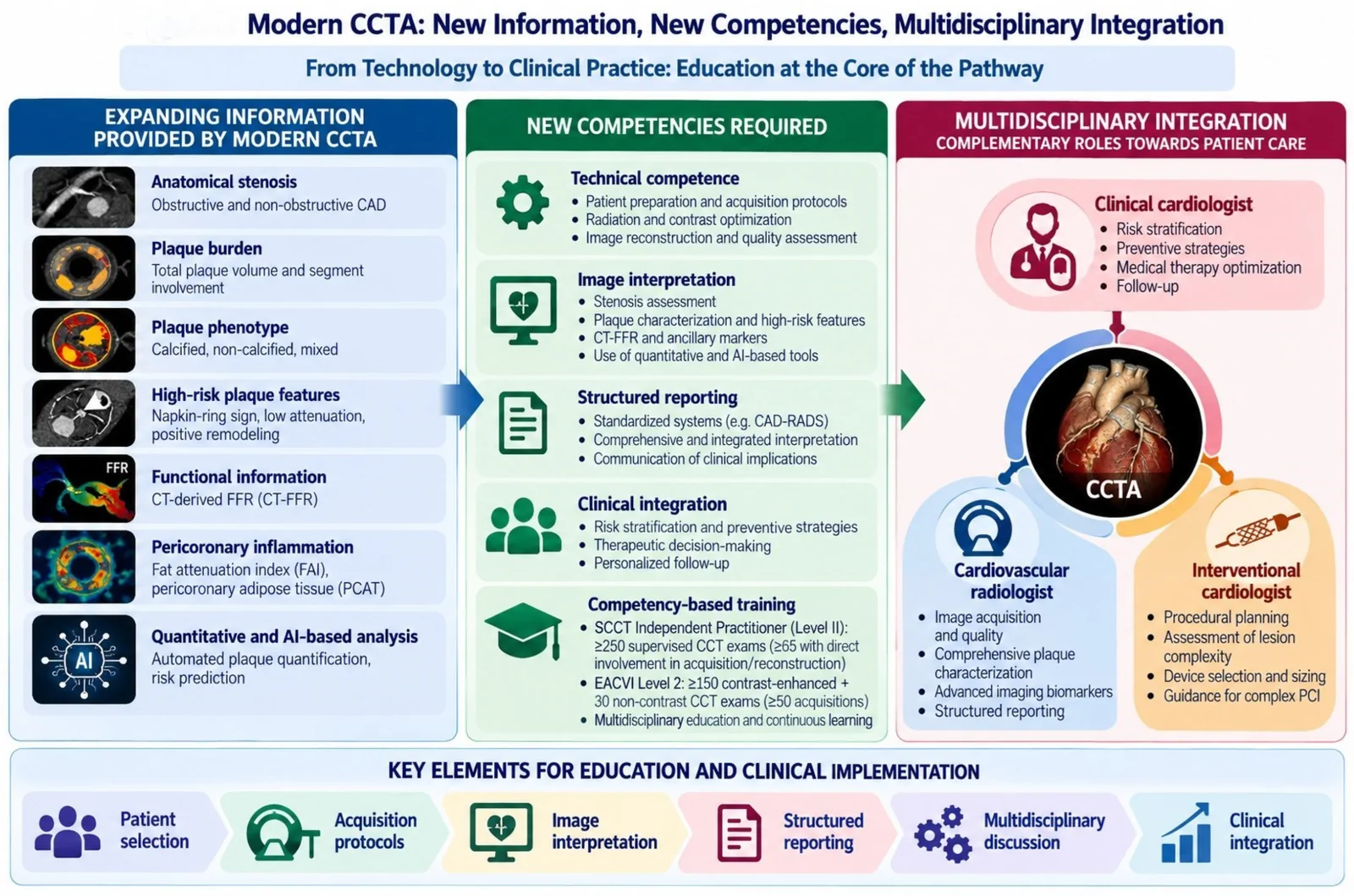
A summary overview of the key features of modern CCTA, which combines the information provided by the technique with the necessary expertise and multidisciplinary integration. CCTA, Coronary computed tomography angiography; CAD, coronary artery disease; CT-FFR, computed tomography-derived fractional flow reserve; FAI, fat attenuation index; PCAT, pericoronary adipose tissue; CAD-RADS, Coronary Artery Disease Reporting and Data System; SCCT, Society of Cardiovascular Computed Tomography; EACVI, European Association of cardiovascular imaging; PCI, percutaneous coronary interventions.

While technological advances have driven the expansion of CCTA, training currently represents one of its greatest challenges. The clinical value of modern CCTA depends not only on technology but also on the ability to accurately interpret imaging findings and integrate them into patient management. As CCTA evolves into a comprehensive assessment of coronary atherosclerosis, technological innovation must be accompanied by the development of new professional skills. The information provided by CCTA is relevant to multiple specialists, including both clinical and interventional cardiologists, as well as cardiovascular radiologists, making multidisciplinary collaboration increasingly essential (Figure 3).

It is important to emphasize that multidisciplinary training must be supported by measurable competency standards. The SCCT competency framework for Independent Practitioners (Level II) requires supervised experience involving at least 250 CCTA examinations, including direct involvement in patient preparation, image acquisition, and image reconstruction in at least 65 cases [75]. Similarly, EACVI Level 2 certification requires the interpretation of at least 150 CCTA exams with contrast and 30 without contrast, with active involvement in the acquisition of at least 50 cases [76]. Although these frameworks differ in their specific requirements, they provide established competency-based models that CCTA centers can adopt in accordance with national accreditation requirements. It is important to emphasize that procedural volume alone does not attest to competence and should be supplemented by training in patient selection, acquisition protocols, radiation and contrast agent optimization, image interpretation, and appropriate follow-up clinical management.

For the clinical cardiologist, CCTA takes on a significance that goes beyond the diagnostic test, becoming a tool for cardiovascular risk stratification and prevention. Familiarity with standardized reporting systems, such as CAD-RADS, i.e., Coronary Artery Disease-Reporting and Data System [77], together with the interpretation of the plaque phenotype and non-obstructive CAD, enables the identification of patients who could benefit from more intensive preventive strategies, optimization of lipid-lowering therapy, novel pharmacological treatments, and personalized follow-up.

For the interventional cardiologist, CCTA has become an increasingly valuable tool for procedural planning [78,79,80]. Three-dimensional visualization of the coronary tree allows for accurate assessment of lesion length, vessel diameter, coronary calcification, tortuosity, bifurcation anatomy, and chronic total occlusions, facilitating the selection of the optimal interventional strategy prior to catheterization. CCTA may also guide device selection, stent sizing, and the identification of lesions requiring specific techniques, such as atherectomy or intravascular lithotripsy.

Finally, the role of the cardiovascular radiologist has also evolved, going beyond the simple anatomical description of coronary stenosis. Modern reporting should include comprehensive characterization of coronary atherosclerosis, requiring expertise in plaque pathophysiology, high-risk plaque features, prognostic implications, and standardized reporting systems [81]. The integration of emerging technologies—including CT-derived fractional flow reserve (CT-FFR), automated plaque quantification, and artificial intelligence—will further expand these competencies.

Multidisciplinary training programs should become an integral component of CCTA implementation. Ultimately, the educational challenge extends far beyond learning how to perform, report or interpret a CCTA examination. It involves understanding how to translate its multidimensional information into more effective cardiovascular prevention, better therapeutic decision-making, and personalized patient care.

Although universal screening is neither realistic nor sustainable, targeted use of CCTA may be considered in selected clinical situations. In asymptomatic individuals, however, CAC should remain the preferred imaging approach for the most accurate risk assessment, while CCTA should be reserved for selected cases in which further anatomical characterization is expected to influence clinical management. Uncertainty regarding risk should be defined based on proximity to the treatment decision threshold and the presence of relevant risk-modifying factors, including a family history of early myocardial infarction and elevated lipoprotein(a) levels.

Healthcare policies should also promote integrated diagnostic pathways involving cardiologists, radiologists, and general practitioners. The feasibility of incorporating CCTA as a first-line diagnostic strategy has already been proposed within the healthcare system. In the United Kingdom, the NICE CG95 guideline recommends CCTA as a first-line diagnostic test for patients with recent-onset stable chest pain when clinical evaluation suggests typical or atypical angina, providing a well-established example of guideline-driven integration of CCTA into diagnostic pathways and resource allocation [82]. Current organizational and reimbursement models should evolve beyond the traditional view of CCTA as a purely anatomical test for detecting coronary stenosis and recognize its broader diagnostic, prognostic, and preventive value.

### 3.7. Future Development

CCTA is entering a new phase driven by four major innovations: photon-counting CT, AI, assessment of coronary inflammation through the fat attenuation index (FAI), and integration of functional information with CT-derived fractional flow reserve (CT-FFR).

Photon-counting CT is one of the most promising advances in cardiovascular imaging [83,84,85]. Compared with conventional CT, it provides higher spatial resolution, improved contrast-to-noise ratio, and superior tissue characterization, with the potential to reduce calcium blooming artifacts, improve visualization of small coronary vessels, and enhance plaque characterization. However, its incremental clinical value over contemporary energy-integrating detector CT, particularly in terms of patient outcomes and cost-effectiveness, remains to be established.

AI is expanding the role of CCTA through automated quantitative plaque analysis [86,87,88]. AI-based algorithms enable rapid and reproducible assessment of plaque burden and composition, reducing interobserver variability and facilitating standardized reporting. AI-based quantitative plaque analysis is nearing clinical implementation, and several commercial platforms have obtained regulatory approval, including FDA-cleared software for quantitative coronary plaque assessment. Selected algorithms have shown good agreement with expert readers and with invasive gold standards such as IVUS, supporting their technical validity [89]. However, regulatory clearance should not be interpreted as evidence of improved clinical outcomes. Much of the published evidence remains retrospective, and external prospective validation across independent populations, scanner manufacturers, acquisition protocols, and clinical settings is still limited. Furthermore, reproducibility across serial examinations and clinically significant thresholds for plaque progression require further definition.

Another emerging application is the evaluation of coronary inflammation using the FAI [90,91,92]. By assessing pericoronary adipose tissue, FAI provides a non-invasive marker of vascular inflammation capable of integrating plaque burden and plaque phenotype, offering a more comprehensive assessment of cardiovascular risk.

It is important to emphasize that the ORFAN study demonstrated that information on perivascular inflammation derived from CCTA provided incremental prognostic stratification—including patients without obstructive CAD—supporting the concept that cardiovascular risk is determined not only by luminal stenosis but also by the biological activity of the underlying atherosclerotic process [93]. However, these findings remain prognostic and observational in nature and do not demonstrate that modifying preventive therapy based on the FAI improves clinical outcomes. Furthermore, FAI measurements are sensitive to technical factors, including tube voltage, scanner characteristics/manufacturer, and reconstruction algorithms, which can influence attenuation values and limit comparability across centers and between serial examinations [94]. Standardization of acquisition, reconstruction, and analysis protocols is therefore necessary before FAI can be widely implemented as a reproducible clinical biomarker.

The integration of functional assessment represents a further step in the evolution of CCTA. Among available techniques, CT-FFR is currently the most extensively validated [95]. By combining anatomical imaging with computational fluid dynamics, CT-FFR improves identification of hemodynamically significant coronary lesions and may reduce unnecessary ICA. Despite this progress, their implementation in routine clinical practice requires further validation. Most of the data supporting CT-FFR derives from symptomatic patients with suspected CAD [96,97,98], while robust data in primary prevention and asymptomatic populations remain limited. Likewise, photon-counting CT, AI-based quantitative plaque analysis, and FAI require additional standardization and integration into clinical pathways. The future challenge will consist not only in generating a greater volume of imaging information but also in translating these data into clinically meaningful and cost-effective decisions that could improve patient outcomes.

Taken together, contemporary CCTA can provide complementary information across multiple pathobiological domains, ranging from anatomical disease burden and plaque phenotype to lesion-specific functional significance and emerging markers of the coronary metabolic-inflammatory milieu. However, these parameters differ substantially in their degree of validation and guideline endorsement (Table 2).

## 4. Limitations

This perspective represents a narrative, non-systematic synthesis of the literature and does not include a formal systematic review, an evidence-grading process, or a consensus methodology. It is clear that the strength of the evidence varies depending on the CCTA applications discussed. Although the diagnostic and prognostic value of CCTA is well established in symptomatic patients, emerging applications—including AI-based plaque quantification, pericoronary inflammatory imaging, serial assessment of plaque response to therapy, and CCTA-guided prevention—require further standardization and prospective validation. In particular, changes in plaque burden or composition observed in serial CCTA scans do not constitute validated surrogate endpoints for cardiovascular events, and there is currently a lack of evidence from randomized trials demonstrating that CCTA-guided modification of preventive therapy improves clinical outcomes. However, the added value and the novelty of this perspective, shared within a multidisciplinary group, is to synthesize the many different aspects underlying CCTA and to raise awareness among the cardiovascular clinical and scientific community regarding the numerous and varied implications involved.

## 5. Conclusions

The novelty of this perspective lies in highlighting the need to consider the multiple, constantly evolving aspects that characterize the use of CCTA. Beyond guidelines and position statements, every clinician should adopt a broader view and develop an increasing awareness of the balance required in the diagnostic and therapeutic decision-making process.

CCTA represents a comprehensive imaging modality for the assessment of coronary atherosclerosis, providing information on plaque burden, composition, and vulnerability. It integrates traditional clinical risk scores and CAC, thus enabling more accurate cardiovascular risk stratification and personalized preventive strategies.

In symptomatic patients, its role is better established, as randomized evidence, including SCOT-HEART, supports the clinical value of a CCTA-based diagnostic strategy These findings should not simply be extrapolated to CCTA-based screening or plaque-guided prevention in asymptomatic populations. However, by providing information on plaque burden, composition, and high-risk features, CCTA may improve risk characterization beyond luminal stenosis alone. This perspective requires randomized studies and robust evidence to enable effective and system-wide sustainable prevention. Emerging technologies—including photon-counting CT, AI-based plaque analysis, percoronary inflammatory imaging, and CT-FFR—may further expand the information provided by CCTA, again subject to appropriate validation and standardization. The full realization of the CCTA’s potential will depend not only on the technology industry but also on the standardization of appropriate technologies, the development of expertise and multidisciplinary cardiovascular teams, innovation in health policies that promote VBHC and appropriate prescribing in line with the “One Health” view, and equitable access to care through a fair reimbursement policy.

## Figures and Tables

**Table 1:** Qualitative framework for high-quality CCTA [66,67].

Domain	Key Components	Clinical/Technical Purpose
Technology	Last-generation multidetector CT with adequate spatial and temporal resolution; Advanced reconstruction techniques; Photon-counting CT	Diagnostic visualization of coronary anatomy and stenosis; Limitation of artifacts and radiation exposures
Patient preparation	Assessment of HR and rhythm; HR optimization; Breath-hold preparation; Assessment of renal function and contrast allergy	Optimize image quality and reduce non-diagnostic examinations
Acquisition and reconstruction	Patient- and scanner-specific acquisition protocol; prospective ECG triggering; individualized tube voltage/current; thin-slice reconstruction; optimized contrast injection	Improvement of image quality using the lowest reasonably achievable radiation and contrast exposure
Coronary Vasodilation	Sublingual nitrates if appropriate	Improved coronary lumen visualization
Image analysis	Assessment of stenosis, plaque burden and composition, high-risk plaque; quantitative and AI-assisted tools when appropriately validated	Extend interpretation beyond luminal stenosis and improve reproducibility
Reporting	Structured reporting; CAD-RADS 2.0 when applicable; Integration of stenosis, plaque burden/high-risk features, and functional information when available	Standardize communication; Reduction of inappropriate downstream testing
Expertise and integration	Appropriate CCTA training; Interaction among cardiovascular radiologists, clinical and interventional cardiologists	Technically appropriate acquisition; Clinically contextualized interpretation
Quality and Safety	ALARA principle; Assessment of beta-blocker contraindications; Renal function and iodinated-contrast risk; Nitrates avoidance when contraindicated, particularly with recent phosphodiesterase-5 inhibitor use; Quality-control and audit processes	Minimal radiation exposure; Pharmacological and contrast-related harms; Maintenance of consistent examination quality

CCTA, coronary computed tomography angiography; CT, computed tomography; HR, heart rate.; CAD-RADS, coronary artery disease-reporting and data system.

**Table 2:** Multiparametric pathobiological information of CCTA [17,67,82].

Pathobiological Domain	CCTA-Derived Assessment	Potential Clinical Relevance	Current Guidelines
Coronary anatomy and atherosclerotic burden	Presence and extent of obstructive and non-obstructive CAD Stenosis severity Distribution and overall plaque burden	CAD diagnosis Exclusion of obstructive disease Risk stratification Identification of non-obstructive atherosclerosis to manage preventive management	CCTA is recommended as a first-line non-invasive anatomical test in selected patients with suspected CCS CCTA is particularly effective for ruling out obstructive CAD and detecting non-obstructive atherosclerosis
Plaque morphology and vulnerability	Calcified, non-calcified, mixed plaque Low-attenuation plaque Positive remodeling Napkin-ring sign Spotty calcification	Prognostic information beyond stenosis assessment Characterization of coronary atherosclerosis	ESC guidelines recognize the prognostic relevance of adverse plaque features and support comprehensive CCTA reporting, while also highlighting technical limitations None of the current guidelines recommend basing treatment decisions solely on the high-risk plaque phenotype
Functional significance of coronary lesions	CT-FFR	Identification of anatomically intermediate lesions with functional significance Avoidance of unnecessary ICA Indication to further testing or revascularization	CT-FFR can complement CCTA when the functional significance of a stenosis is uncertain CT-FFR is particularly indicated for intermediate stenoses, provided that image quality is adequate
PCAT/coronary inflammation	PCAT attenuation FAI and related perivascular inflammatory biomarkers	Incremental prognostic information, including prediction of cardiac events beyond conventional anatomical assessment	Prognostic evidence is increasing, however routine FAI/PCAT-guided clinical management is not currently recommended Technical standardization and clinical utility must be confirmed and defined

CCTA, coronary computed tomography angiography; CAD, coronary artery disease; CCS, chronic coronary syndrome; CT-FFR, computed tomography-fractional flow reserve; PCAT, pericoronary adipose tissue; FAI, fat attenuation index.

## Data Availability

No new data were created or analyzed in this study. Data sharing is not applicable to this article.

## Abbreviations

The following abbreviations are used in this manuscript:

CCTA	coronary computed tomography angiography
ICA	invasive coronary angiography
CAD	coronary artery disease
CAC	coronary artery calcium score
CT	computed tomography
VBHC	value-based health care
FFR	fractional flow reserve
CAD-RADS	coronary artery disease-reporting and data system
FAI	fat attenuation index
AI	artificial intelligence
IVUS	intravascular ultrasound
